# Construction of a Prognostic Model of Prostate Cancer Based on Immune and Metabolic Genes and Experimental Validation of the Gene AK5

**DOI:** 10.32604/or.2025.066783

**Published:** 2025-10-22

**Authors:** Wenjie Zhou, Jiawei Ding, Danfeng Xu

**Affiliations:** Department of Urology, Ruijin Hospital, Shanghai Jiao Tong University School of Medicine, Shanghai, 200025, China

**Keywords:** prognostic model, prostate cancer, immune, metabolism

## Abstract

**Objectives:**

Despite the fact that prostate cancer is one of the most common tumors in men, this study intends to evaluate the predictive significance of immune and metabolic genes in prostate cancer using multi-omics data and experimental validation.

**Methods:**

The research developed and validated a prognostic model utilizing The Cancer Genome Atlas (TCGA) and Gene Expression Omnibus (GEO) databases, integrating immune and metabolic gene sets. Additionally, the prognostic gene Adenylate Kinase 5 (AK5) was analyzed in prostate cancer tissue microarrays from Ruijin Hospital. The functional role of the AK5 gene was validated through knockdown and overexpression experiments in four prostate cancer cell lines, employing cell proliferation assays, colony formation assays, and both xenograft models in nude mice and patient-derived xenograft models.

**Results:**

This research developed a prognostic model comprising ten genes, which was validated across multiple datasets for its predictive efficacy. Experimental results indicated that AK5 is significantly expressed in prostate cancer and facilitates tumor proliferation; knockdown of AK5 inhibited cell colony formation and growth of subcutaneous xenografts in nude mice, while AK5 inhibitors significantly reduced tumor volume in patient-derived xenografts.

**Conclusion:**

This study constructed a prognostic model with clinical potential and preliminarily confirmed the oncogenic role of AK5 in prostate cancer. The findings indicate that focusing on the immunological metabolic axis and the AK5 gene may offer novel approaches for prostate cancer treatment.

## 1 Introduction

In most regionsof the world, prostate cancer (PCa) has become a major malignant tumor among men [1–3]. According to a study conducted in the United States, the incidence of prostate cancer accounted for 29% of the total cancer diagnoses among men in 2023, exhibiting an annual increase of 3% from 2014 to 2019, thereby demonstrating a rising trend year by year [4]. Currently, radiotherapy and surgical resection are the primary treatment modalities for locally advanced prostate cancer [5,6], demonstrating promising results as they continue to be optimized. With advancements in diagnostic and therapeutic approaches, the overall survival (OS) rate of prostate cancer patients has been on the rise [7]; nevertheless, it continues to be one of the leading causes of male cancer-related mortality, and the progression-free survival (PFS) rate for these patients is concerning [8,9]. Androgen deprivation therapy (ADT) is the major treatment for non-metastatic prostate cancer [10,11]; however, large number of patients ultimately progress to castration-resistant prostate cancer (CRPC) [12], posing a formidable challenge to treatment [13].

Alterations in immunological and metabolic genes are intricately associated with the advancement of prostate cancer [14,15]. Nowadays, as high-throughput sequencing methods have improved, a wealth of biological information related to prostate cancer has been continuously published and refined [16–19]. This has provided significant resources for integrating clinical data, particularly regarding patient prognosis, to identify novel prognostic biomarkers for prostate cancer. Previous studies have effectively validated and assessed prognostic models for prostate cancer constructed using immune cell-related gene sets derived from transcriptomic data [20].

Based on the immune and metabolic genes, this study aims to establish a prognostic model through the multi-omics data, and validate it through experiments.

## 2 Materials and Methods

### 2.1 Acquisition of Immune and Metabolic Gene Sets

The ImmPort database (https://www.immport.org/shared/genelists) (accessed on 01 November 2024) provided the immune gene set, which includes various genes associated with human immune cells and immune responses. In total, 2914 immune genes were extracted. The MSigDB database served as the source for the metabolic gene set on the Gene Set Enrichment Analysis (GSEA) official website (https://www.gsea-msigdb.org/gsea/msigdb) (accessed on 01 November 2024), comprising genes related to human metabolic pathways. In total, 829 metabolic genes were extracted, encompassing the metabolism of numerous substances in the human body.

### 2.2 Acquisition of PCa Data from Public Databases

Transcriptomic data for prostate cancer patients and clinical features were obtained from TCGA (https://portal.gdc.cancer.gov/) (accessed on 01 November 2024). After processing with Strawberry Perl version 5.30.0.1 self-written scripts, a total of 497 tumor samples containing relevant clinical characteristics were identified with clinical details. Additionally, mutation data for all downloaded samples were processed to obtain mutation information for each gene and the tumor mutational burden (TMB) for each sample. This study also collected gene expression profiles and clinical data from 248 samples in the GSE116918 dataset from GEO (https://www.ncbi.nlm.nih.gov/geo) (accessed on 01 November 2024). Furthermore, single-cell transcriptomic data were selected from the GSE137829 dataset, while the GSE30174 dataset was utilized for single-gene differential expression analysis.

### 2.3 Differential Analysis and Extraction of Differential Gene Expression Levels

Extraction of gene expression levels was performed through the “limma” (v3.62.2) and “sva” (v3.54.0) R packages. To conduct differential analysis, the “limma” package was used to calculate the logFC and *p*-value. The screening criteria included an absolute value of logFC greater than 1 and a False Discovery Rate (FDR) value less than 0.05. The “ggplot2” package (v3.5.2) was used to create volcano plot and the “pheatmap” package (v1.0.12) was employed for creating heatmap.

### 2.4 Non-Negative Matrix Factorization (NMF) Subtyping

NMF was performed to conduct molecular subtyping analysis of tumor samples. Genes significantly associated with patients’ progression-free interval (PFI) were screened through the univariate COX regression model (*p* < 0.01). Subsequently, the best number of subtypes (3) was decided by calculating the cophenetic coefficient through multi-k value validation (k = 2–10). The clustering structure was visualized through the consensus matrix heatmap.

### 2.5 Analysis of the Tumor Microenvironment (TME)

The study used the “limma” package to read and filter gene expression data from TCGA prostate cancer samples and the “estimate” package (v1.0.13) was employed to filter out common genes associated with the TME from the expression data. Tumor purity, stromal score, and immune score were measured through “estimateScore” function. The “reshape2” package (v1.4.4) was utilized to process the TME scoring data and grouping data, while the “ggpubr” package (v0.6.0) was employed to create combined violin and box plots.

### 2.6 Immune Cell Analysis

The study used the “MCPcounter” package (v1.2.0) to quantify the infiltration of 2 types of stromal cells and 8 types of immune cells in TME. The infiltration scores for various immune cell types in the sample were computed. The tumor microenvironment scores and immune cell infiltration data were integrated, and the “pheatmap” package was utilized to create heat maps of immune cells based on various types or groups.

### 2.7 Construction of a Prognostic Model

This step primarily utilizes the following R packages: the “survival” package (v3.8.3) for survival analysis, the “caret” package (v7.0.1) for data partitioning, the “glmnet” package (v4.1.8) for performing Least Absolute Shrinkage and Selection Operator (LASSO) regression, and the “survminer” (v0.5.0) and “timeROC” packages (v0.4) for the visualization of survival curves and time-dependent Receiver operating characteristic (ROC) analysis. Initially, a dataset comprising 497 prostate cancer samples from TCGA and 248 samples from GEO is organized, converting progression-free survival time from days to years. Subsequently, a random model training is conducted, utilizing the “createDataPartition” function to split the TCGA data into training and testing sets with a ratio of 7:3. Univariate Cox regression is performed for each gene in the training group to filter significant genes (*p* < 0.05), retaining candidate genes associated with progression-free survival. Then we conducted LASSO regression to select genes and cross-validation to determine the best λ value, thereby eliminating redundant genes and generating a list of key genes. 10-fold cross-validation was used in the LASSO regression. The value of λ that gives the minimum cross-validated error was selected as the optimal λ. A multivariable Cox model is subsequently developed utilizing the genes identified through LASSO and stepwise regression is utilized to optimize the model, retaining independent prognostic factors. The “predict” function is used to calculate risk scores for each dataset. The equation for determining the risk score is $Risk\;Score=\sum_{i=1}^{n}(Coef_{i}\times Expression_{i})$, where $Coef_{i}$ denotes the regression coefficient of the *i*th gene in the multivariable Cox model, $Expression_{i}$ denotes the expression value of the *i*th gene (standardized value), and *n* denotes the number of genes retained in the model.

### 2.8 Validation of Prognostic Model

The validation process includes the assessment of survival differences, employing the Log-rank test via the “survdiff” function to find out the significance of progression-free survival differences among groups and to generate survival curves. The model validation encompasses internal validation, which involves calculating the area under the ROC curve (AUC) for both the training and testing sets to evaluate the model’s predictive performance for progression-free survival at 1, 3, and 5 years; and external validation, which utilizes GEO data to verify the model’s generalizability and guarantee the dependability of the findings.

### 2.9 Independent Prognostic Analysis

The “survival” package was utilized to assess the independence of clinical variables, including T stage, N stage, age, and risk scores, as prognostic factors. This was accomplished using both univariate and multivariate Cox regression analyses, complemented by the visualization of results via forest plots. The “bioForest” function was employed to generate the forest plots to illustrate the Cox regression outcomes.

### 2.10 Construction of a Nomogram

This study integrates clinical data and risk scores to construct nomograms with the use of the Cox proportional hazards model, followed by calibration and validation. The nomogram was developed using the following R packages: the “survival” package for survival analysis, the “regplot” package (v1.1) for nomogram plotting, and functions such as cph and calibrate from the “rms” package (v8.0.0) for model calibration. The calibration curves evaluate the model’s predictive accuracy at 1-year, 3-year, and 5-year intervals. The consistency index (C-index) quantifies the discriminatory capability of the model.

### 2.11 Analysis of the Correlation between Risk Scores and Immune Cells

It is mainly accomplished with the help of the “limma”, “ggpubr”, and “corrplot” (v0.95) packages. The integration of risk scores and immune cell infiltration data allows for a systematic evaluation of the differences in the immune microenvironment between groups and their associations with the risk scores.

### 2.12 Gene Set Enrichment Analysis (GSEA)

The study examined how biological characteristics and pathways varied throughout groups through the “limma”, “org.Hs.eg.db” (v3.20.0), “clusterProfiler” (v4.14.6), and “enrichplot” (v1.26.6) packages. GSEA was performed utilizing Gene Ontology (GO) and the Kyoto Encyclopedia of Genes and Genomes (KEGG) to compare high-risk and low-risk groups.

### 2.13 Correlation of Tumor Mutation Burden with Survival Analysis

The research incorporated multi-omics data, encompassing TMB, microsatellite instability (MSI), immune cell infiltration, and risk scores. The Pearson correlation coefficient was utilized to compute the correlation matrix among TMB, MSI, risk scores, and the extent of immune cell infiltration. A chord diagram was generated using the “circlize” package (v0.4.16). In conducting survival analysis of TMB, the “survival” package and the “survminer” package are primarily utilized, with the former employed for the processing and analysis of survival data, while the latter is used for the visualization of results.

### 2.14 Sources of Clinical Samples and Tissue Microarrays

The research employed a tissue microarray (TMA) consisting of 72 prostate cancer specimens sourced from radical prostatectomies conducted at Ruijin Hospital. Relevant clinical information of the patients was collected. Fresh tissue samples for xenografting were acquired from the operating room of the hospital. H-scores of AK5 were determined by two blinded pathologists and were generated by calculating the sum of the average percent positive multiplied by immunohistochemistry staining intensity (0–3). The tissue microarray was manufactured by Shanghai Ruchuang Biotechnology Co., Ltd. (Shanghai, China). The research was conducted in accordance with the ethical principles outlined in the Declaration of Helsinki, along with approval from the Ruijin Hospital Ethics Committee, Shanghai Jiao Tong University School of Medicine, the approval number of which is No. 2024-102. All of the study’s individual participants provided written informed consent. Participants were made aware of the goals, methods, any dangers, and their freedom to leave the study at any time.

### 2.15 Bioinformatics Analysis Related to AK5

The TCGA dataset and GEO dataset GSE30174 were utilized to analyze the expression differences of AK5 between tumor and normal tissues. In survival analysis, tumor patients were classified into high-expression and low-expression groups according to the median levels of AK5 expression. Univariate and multivariate Cox regression analyses were conducted in the prostate cancer cohort at Ruijin Hospital. Variables such as age, Gleason score, and clinical stage were screened to analyze whether AK5 expression has independent prognostic value. In addition, the expression distribution of AK5 at the single-cell level and the expression levels of AK5 in different cell types were plotted on the basis of the GSE137829 dataset of GEO. In the analysis, “ggplot2” package was used to create pie chart and “Seurat” package (v5.3.0) was used to create umap.

### 2.16 Cell Lines

The prostate cancer cell lines VCaP (CL-0241), LNCaP (CL-0143) and DU145 (CL-0075), and the 293T (CL-0005) cell line were purchased from Pricella Biotechnology Co., Ltd. (Wuhan, China), while the MDAPCa2b (YS1269C) cell line was acquired from Yaji Biological Co., Ltd. (Shanghai, China). All of these cell lines were free of mycoplasma contamination and identified by Short Tandem Repeat. The MDAPCa2b cell line was cultured in the medium (YS1730M) purchased from Yaji Biological Co., Ltd. (Shanghai, China) containing F-12K medium, 20% Fetal Bovine Serum (FBS), 25 ng/mL cholera toxin, 10 ng/mL mouse epidermal growth factor, 0.005 mM phosphoethanolamine, 100 pg/mL hydrocortisone, 45 nM sodium selenite and 0.005 mg/mL human recombinant insulin. The VCaP and 293T cell lines were cultured in Dulbecco’s Modified Eagle’s Medium (DMEM, PM150210, Pricella Biotechnology) supplemented 1% penicillin/streptomycin (PB180120, Pricella Biotechnology) and 10% FBS (164210, Pricella Biotechnology), while the DU145 cell line was maintained in Minimum Essential Medium (MEM, PM150410, Pricella Biotechnology) containing 10% FBS (164210, Pricella Biotechnology) and 1% penicillin/streptomycin (PB180120, Pricella Biotechnology). The LNCaP cell line was cultured in Roswell Park Memorial Institute-1640 (RPMI-1640, PM150110, Pricella Biotechnology) medium supplemented with 10% FBS (164210, Pricella Biotechnology) and 1% penicillin/streptomycin (PB180120, Pricella Biotechnology). All four cell lines were incubated under conditions of 37°C, 5% CO_2_, and 95% humidity.

### 2.17 Plasmid Transfection

The study applied pLKO.1 vector (Addgene, Watertown, MA, USA) to construct a specific AK5 shRNA-expressing plasmid. The constructed pLKO.1-shAK5 plasmid was transfected into prostate cancer cells using Lipofectamine 3000 Transfection Reagent (Thermo Fisher, L3000015, Waltham, MA, USA). DNA-Lipofectamine complexes were formed in Opti-MEM medium (Thermo Fisher, 31985062) and added to cells. After 24–48 h, transfected cells were selected with puromycin (Thermo Fisher, A1113803) to establish stable shRNA-expressing populations. We selected 2 target sequences of the shRNA constructs. The sequences were as follows: non-target-shRNA forward primer: 5^′^-CCGGCAACAAGATGAAGAGCACCAACTCGAGTTGGTGCTCTTCATCTTGTTGTTTTTG-3^′^ and reverse primer: 5^′^-AATTCAAAAACAACAAGATGAAGAGCACCAACTCGAGTTGGTGCTCTTCATCT TGTTG-3^′^. shAK5#1 forward primer: 5^′^-CCGGTGATGCATTTCAGCAATTATCTCGAGATAATTGCT GAAATGCATCACTTTTTG-3^′^ and reverse primer: 5^′^-AATTCAAAAAGTGATGCATTTCAGCAATTAT CTCGAGATAATTGCTGAAATGCATCAC-3^′^. shAK5#2 forward primer: 5^′^-CCGGCGATATGGATTCCA ATACATTCTCGAGAATGTATTGGAATCCATATCGTTTTTG-3^′^ and reverse primer: 5^′^-AATTCAAAA ACGATATGGATTCCAATACATTCTCGAGAATGTATTGGAATCCATATCG-3^′^.

### 2.18 Reverse Transcription Quantitative Polymerase Chain Reaction (RT–qPCR)

The study used TRIzol reagent (Thermo Fisher, 15596026CN, Waltham, MA, USA) to extract total RNA from the cells. Reverse transcription was performed with a PrimeScript RT kit (Takara Bio, RR047A, Shiga, Japan) and RT–qPCR was performed using a real-time quantitative PCR instrument (Applied Biosystems 7500 Fast Real-Time PCR System, Thermo Fisher) and TB Green PreMix Ex Taq (RR420A, Takara Bio). AK5 forward primer, 5^′^-TCTAAGCCCGAAGATCCAGTAG-3^′^. AK5 reverse primer, 5^′^-GTGACTGTCCTCCATTTAGTGG-3^′^. GAPDH forward primer 5^′^-GAAGGTGAAGGTCGGAGTC-3^′^, GAPDH reverse primer 5^′^-GAAGATGGTGATGGGATTTC-3^′^. The relative expression of AK5 was calculated using the 2^−ΔΔCt^ method.

### 2.19 3-(4,5-dimethylthiazol-2-yl)-5-(3-carboxymethoxyphenyl)-2-(4-sulfophenyl)-2H-tetrazolium (MTS) Cell Proliferation Assay

The VCaP and MDAPCa2b cell lines were established with shCtrl (non-targeting control), shAK5#1, and shAK5#2, while the LNCaP and DU145 cell lines were established with EV (empty vector control) and AK5-OE. Each prostate cancer cell line was seeded into 96-well plates at a density of 2 × 10^3^ to 3 × 10^3^ cells per well (100 μL of culture medium), with six replicate wells per group. The cells were pre-cultured in a 37°C, 5% CO_2_ incubator for 24 h. For the MTS assay, measurements were taken daily over a period of five days, with 20 μL of MTS reagent (Abcam, ab197010, Cambridge, UK) added to each well, mixed gently, and incubated in the dark at 37°C for 2–3 h. Optical Density at 490 nm (OD_490_) was measured using a microplate reader (Multiskan FC Microplate Photometer, 1410101, Thermo Fisher), and the data were recorded. In addition, the VCaP cell line was used to observe the impact of AK5 knockdown on the effects of 5 μM of Apigenin (SANTA CRUZ BIOTECHNOLOGY, sc-3529c, Dallas, TX, USA) on cell proliferation. The LNCaP cell line was used to observe the impact of AK5 overexpression on the effects of 5 μM of Apigenin on cell proliferation.

### 2.20 Colony Formation Assay

The VCaP and MDAPCa2b cell lines were seeded at a density of 500 cells per well in 2 mL of culture medium, while the LNCaP and DU145 cell lines were seeded at a density of 300 cells per well in 2 mL of culture medium. The cells were pre-cultured in a 37°C, 5% CO_2_ incubator for 24 h. All groups were maintained for 10 to 14 days until colonies with a diameter of ≥50 µm were visibly formed. The culture medium was removed, and the wells were rinsed twice with Phosphate Buffered Saline (PBS, 1×, pH 7.4, Servicebio, G4202-100ML, Wuhan, China). Subsequently, 4% paraformaldehyde (G1101-500ML, Servicebio) was added for fixation for 15 min, followed by washing with PBS. A 0.5% crystal violet solution was then applied for 20 min, and the wells were slowly rinsed with deionized water until the background became clear. The plates were allowed to dry at room temperature, and images were photoed for preservation. In addition, in the VCap and MDAPCa2b cell lines, four concentrations of 1, 2.5, 5, and 10 μM of Apigenin were used to observe its effects on colony formation.

### 2.21 Subcutaneous Transplantation of Nude Mice

The research developed a subcutaneous tumor model utilizing the prostate cancer cell line VCaP. Male BALB/c nude mice, aged 4 weeks (provided by the Department of Laboratory Animal Science, Shanghai Jiao Tong University School of Medicine) were selected and were housed under SPF conditions (22 ± 1°C, 50 ± 10% humidity, 12 h light/dark) with corncob bedding, PVC enrichment, and cohorting (2 mice/cage). The experiment consisted of three groups: the EV group, which served as the empty vector control group transfected with an empty vector virus; the shAK5 group, which represented the AK5 gene knockdown group, where AK5 expression was reduced via lentivirus-mediated shRNA; and the recovery group, in which wild-type AK5 gene was re-expressed in the shAK5 cells after knockdown. Each group consisted of 5 biological replicates. Cells in the logarithmic growth phase were harvested and adjusted to a specific density of 1 × 10^7^ cells/mL. 200 μL of cell suspension mixed with Matrigel (Corning, 356234, New York, NY, USA) was subcutaneously injected into the right inguinal region of each nude mouse, followed by gentle massage of the injection site to ensure uniform distribution of the cells. Starting from the fourth day post-inoculation, the tumor volume was calculated every seven days for a duration of five weeks using the formula $V=0.5\times L\times W^{2}$, where V is the volume, L is the longest axis and W is the perpendicular width. The animal experiments were approved by the Institutional Animal Care and Use Committee (IACUC) of Shanghai Jiao Tong University School of Medicine, the approval number of which is JUMC2023-174-B. All procedures adhered to the “Guidelines for the Ethical Review of Animal Welfare in Experiments”. The maximal volume of mouse tumor permitted by the animal ethics committee is 1500 mm^3^. Our study strictly adhered to this standard and the volume of all the subcutaneous tumors in the mice did not exceed 1500 mm^3^.

### 2.22 Patient-Derived Xenograft (PDX) Model

Fresh tumor specimens with a ratio of ≥70% of live cells were selected as transplant material. Each mouse was implanted unilaterally at one site and divided into two groups: the Apigenin group, which received a daily dosage of 100 mg/kg dissolved in Dimenthyl sulfoxide (DMSO, GC203002-100 mL, Servicebio), and the control group, which was administered the same dosage of DMSO. Each group consisted of 8 biological replicates. The percentage change in tumor volume in the PDX model is presented in the form of a bar chart.

### 2.23 Statistical Analysis

All statistical analyses were conducted using R version 4.3.2 (R Foundation for Statistical Computing, Vienna, Austria) and GraphPad Prism version 9 (GraphPad Software, San Diego, CA, USA). In survival analysis, the survival curves were compared using the Log-rank test. Differential gene analysis was conducted using the Wilcoxon rank-sum test, and comparisons between two groups were evaluated with the *t*-test. One-way analysis of variance (ANOVA) was utilized for comparisons among multiple groups. A *p*-value below 0.05 was deemed indicative of statistical significance.

## 3 Results

### 3.1 Construction of Different Subtypes of PCa Based on Differential Expression of Immune and Metabolic Genes

**Figure 1:**
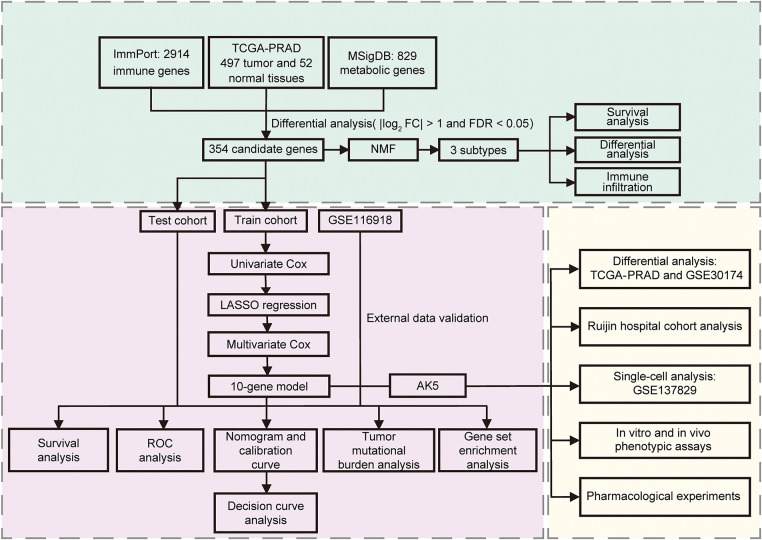
The flowchart of the study. NMF: Non-negative Matrix Factorization; ROC: Receiver operating characteristic

**Figure 2:**
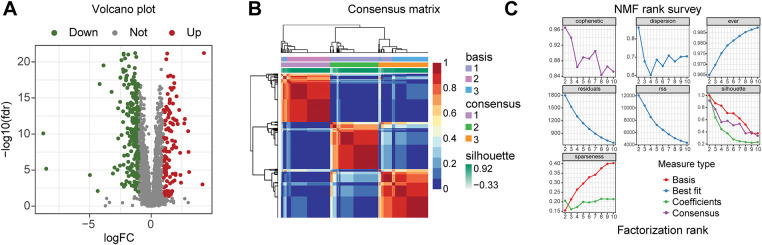

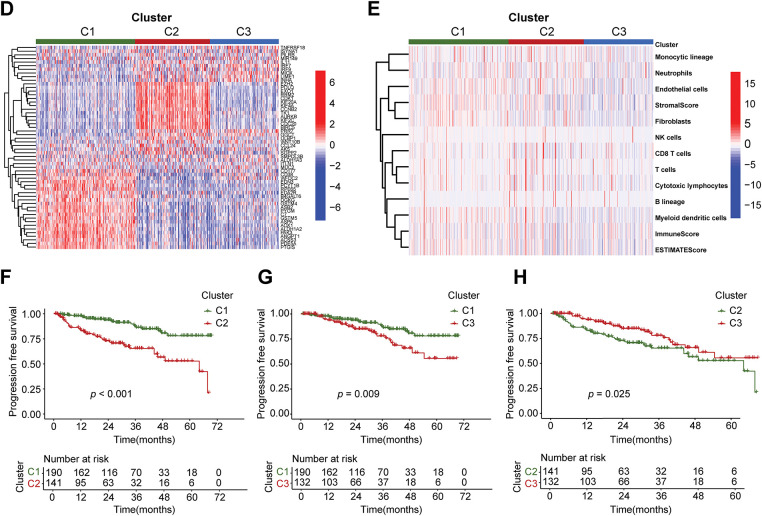
Three subtypes of prostate cancer based on Non-negative Matrix Factorization (NMF) clustering. (**A**) Volcano plot of differential analysis of immune and metabolic genes. The criteria for differential gene filtering are |log2FC| > 1 and FDR < 0.05. (**B**) The consensus matrix heatmap visualizes the clustering structure, with row and column annotations concealed to emphasize the similarity patterns among samples, categorizing prostate cancer samples into three subtypes. (**C**) The NMF typing parameters indicate that the maximum variation of the cophenetic coefficient with respect to k occurs at k = 3, thereby determining the optimal number of clusters to be three. (**D**) The heatmap intuitively illustrates the differential expression patterns of genes across different subtypes. (**E**) The *x*-axis represents three different subtypes, while the *y*-axis illustrates the varying levels of cells and scores. (**F**) The progression-free survival (PFS) of patients with subtype C1 is better than that of those with subtype C2, *p* < 0.001. (**G**) The PFS of patients with subtype C1 is better than that of those with subtype C3, *p* = 0.009. (**H**) The PFS of patients with subtype C3 is better than that of those with subtype C2, *p* = 0.025

The research steps are shown in the flowchart (Fig. 1). The differential analysis of immune and metabolic gene expression levels in TCGA samples indicated significant differences between normal and tumor sample groups (Fig. 2A). Based on these findings, a COX regression analysis was conducted for each gene, identifying significant genes with *p*-values less than 0.01. Subsequently, the NMF algorithm was employed to cluster the filtered data, generating cophenetic plots to evaluate the clustering quality across different k values. The best number of clusters was decided to be the k value with the highest cophenetic coefficient, ultimately resulting in three distinct molecular subtypes for the tumor samples (Fig. 2B,C). There are great distinctions in gene expression among the three distinct subtypes (Fig. 2D), and the progression-free survival of patients across these subtypes also exhibits statistical differences (Fig. 2F–H). The Cluster 1 subtype demonstrates the longest progression-free survival, indicating that the three molecular classifications of prostate cancer established in this study hold considerable significance for predicting patients’ progression-free survival. To further analyze the immunological factors contributing to the prognostic differences among the various subtypes, we explored the differences in immune cell infiltration across these classifications (Fig. 2E). It was observed that in Cluster 1 subtype samples, the infiltration of monocytes, myeloid dendritic cells, neutrophils, and cytotoxic lymphocytes was higher than that in the Cluster 2 and Cluster 3 subtypes, suggesting that the immune cells present in TME may be related to anti-tumor effects.

### 3.2 Construction of a Prognostic Model for PCa Based on Immune and Metabolic Genes

**Figure 3:**
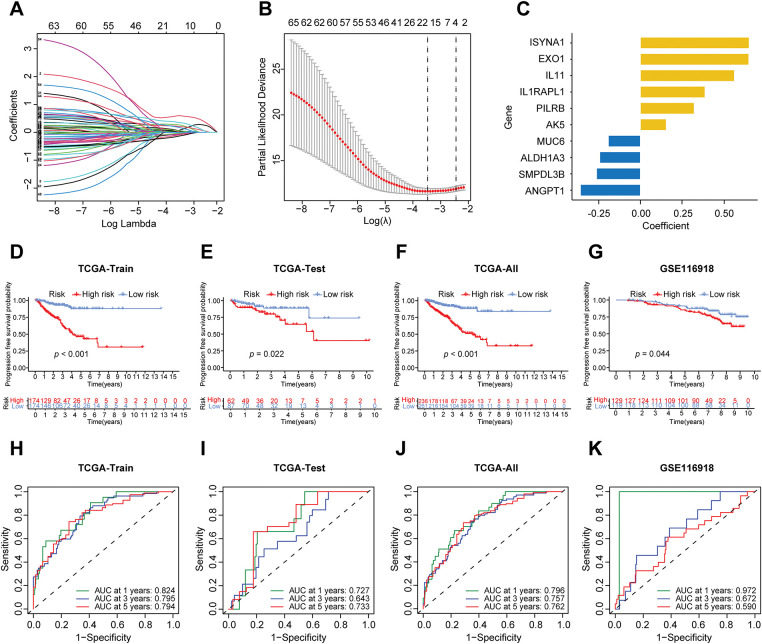
Construction of the prognostic model of prostate cancer. (**A**) The LASSO coefficient path of prognosis-related genes. (**B**) The LASSO regression cross-validation curve further identified 17 prognosis-related genes. (**C**) The multivariable COX stepwise regression ultimately yielded 10 prognostic genes. (**D**) The differences in progression-free survival (PFS) between high-risk and low-risk groups in the TCGA training cohort. (**E**) The differences in PFS between high-risk and low-risk groups in the TCGA testing cohort. (**F**) The differences in PFS between high-risk and low-risk groups in the complete TCGA dataset. (**G**) The differences in PFS between high-risk and low-risk groups in the GEO-GSE116918 dataset. (**H**) ROC curves of the model for predicting 1-, 3-, and 5-year PFS in the TCGA training set. (**I**) ROC curves of the model for predicting 1-, 3-, and 5-year PFS in the TCGA test set. (**J**) ROC curves of the model for predicting 1-, 3-, and 5-year PFS in the complete TCGA dataset. (**K**) ROC curves of the model for predicting 1-, 3-, and 5-year PFS in the GEO-GSE116918 dataset. AUC represents the area under the curve. LASSO: Least Absolute Shrinkage and Selection Operator. AUC: Area under the curve

The advancement of therapeutic strategies has led to a significant improvement in the five-year survival rate for prostate cancer patients [21,22]. To examine the influence of immune and metabolic genes on the prognosis of prostate cancer, progression-free survival (PFS) was selected as the endpoint for the prognostic model. The prior steps entailed the analysis of differential expression data for immune and metabolic genes derived from TCGA samples. The PCa samples from TCGA were randomly allocated into a training set comprising 70% and a testing set comprising 30%. A univariate Cox regression analysis was conducted on the immune metabolic genes from the training set samples, leading to the identification of 65 candidate genes linked to progression-free survival. Subsequently, LASSO regression was applied to the selected genes, and through cross-validation, the optimal λ value was determined, leading to the exclusion of redundant genes and the identification of 17 prognostic-related immune metabolism genes (Fig. 3A,B). A multivariable Cox model was then constructed based on the genes selected by LASSO, utilizing stepwise regression to optimize the model and retain independent prognostic factors, ultimately resulting in the selection of 10 genes for the model (Fig. 3C). The risk score formula was derived based on the expression levels of each gene and their corresponding regression coefficients as follows:

$$\begin{array}{rl} & Risk\;Score=Expression\left(EXO1\right)\times 0.65+Expression(IL11)\times 0.57+Expression(MUC6)\times (-0.19)\\ & +Expression(PILRB)\times 0.32+Expression(IL1RAPL1)\times 0.39+Expression(ANGPT1)\times (-0.36)\\ & +Expression(SMPDL3B)\times (-0.26)+Expression(AK5)\times (0.16)+Expression(ALDH1A3)\\ & \times (-0.24)+Expression(ISYNA1)\times 0.65. \end{array}$$

### 3.3 Validation of the Prognostic Model

A chi-square test was performed to assess significant distribution distinctions in clinical characteristics, including age, T stage, and N stage, between the training and testing sets of PCa samples (Supplementary Table S1). The results showed that all *p* values exceeded 0.05, indicating no statistically significant differences in the distribution of clinical characteristics between the training and testing sets, thus eliminating potential confounding effects of clinical features on model construction.

After constructing the risk score formula, the risk scores of all samples in the TCGA training set, test set, and GEO dataset can be calculated. Using the median risk in the training set as the threshold, all samples can be divided into high-risk and low-risk groups. To verify the effectiveness of the prognostic model, the progression-free survival (PFS) of the high- and low-risk groups was first analyzed in the TCGA training set, test set, total dataset, and GEO validation set, and survival curves were plotted (Fig. 3D–G). A *p*-value less than 0.05 indicates a statistical difference. The results showed that in each dataset, as time passed, the PFS rate of patients in the low-risk group was better than that in the high-risk group. Then, the receiver operating characteristic (ROC) curves of the model for predicting 1-year, 3-year, and 5-year PFS of patients in each dataset were plotted (Fig. 3H–K). It was found that the area under the curve (AUC) was generally above 0.7, especially in predicting 1-year PFS, where the AUC value was the largest. The findings indicate that the risk score model developed in this study demonstrates strong predictive capability.

**Figure 4:**
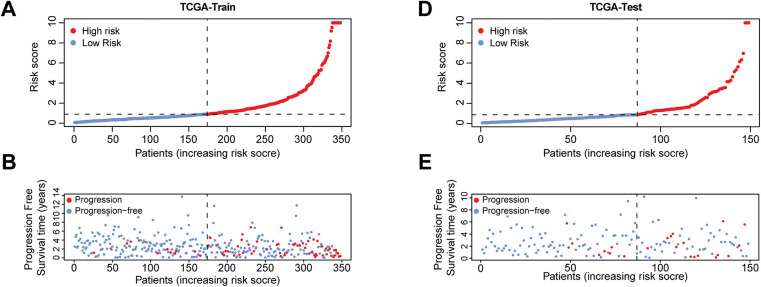

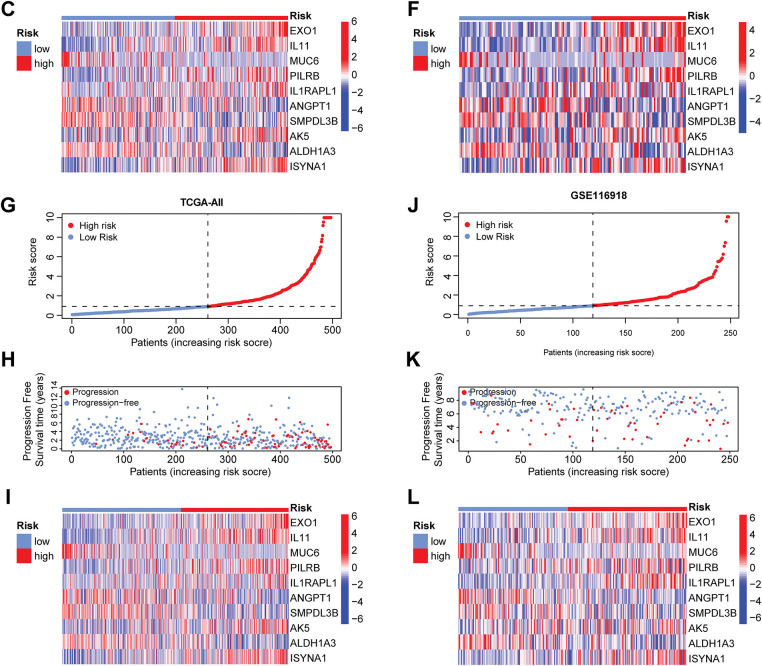
Risk scores, progression-free survival status, and prognostic gene expression profiles of patients in high-risk and low-risk groups across various datasets. The four risk curve plots (**A**, **D**, **G**, **J**) respectively show the changing trends of the risk scores of patients in the high- and low-risk groups in the TCGA training set, test set, complete dataset, and GEO-GSE116918 dataset. The four scatter plots (**B**, **E**, **H**, **K**) respectively show the survival status of patients in the high- and low-risk groups in the TCGA training set, test set, complete dataset, and GEO-GSE116918 dataset. The four gene heat maps (**C**, **F**, **I**, **L**) respectively show the expression differences of 10 prognostic genes used for model construction in patients of the high- and low-risk groups in the TCGA training set, test set, complete dataset, and GEO-GSE116918 dataset

Based on the model, a further in-depth analysis was conducted on the trend of risk score changes, the progression-free survival status, and the differential expression of prognostic genes in the TCGA training set (Fig. 4A–C), test set (Fig. 4D–F), complete dataset (Fig. 4G–I), and GEO-GSE116918 dataset (Fig. 4J–L). The analytical results from multiple datasets indicated that an increase in risk score corresponded with a decrease in the number of patients exhibiting progression-free survival. The ten genes involved in constructing the model exhibited significant expression distinctions between high-risk and low-risk patient groups. Specifically, the expression levels of EXO1, IL11, PILRB, AK5, and ISYNA1 in high-risk samples were found to be higher than those in the low-risk group, suggesting that these genes may be associated with pro-carcinogenic effects.

### 3.4 Construction of Nomogram and Decision Curve

**Figure 5:**
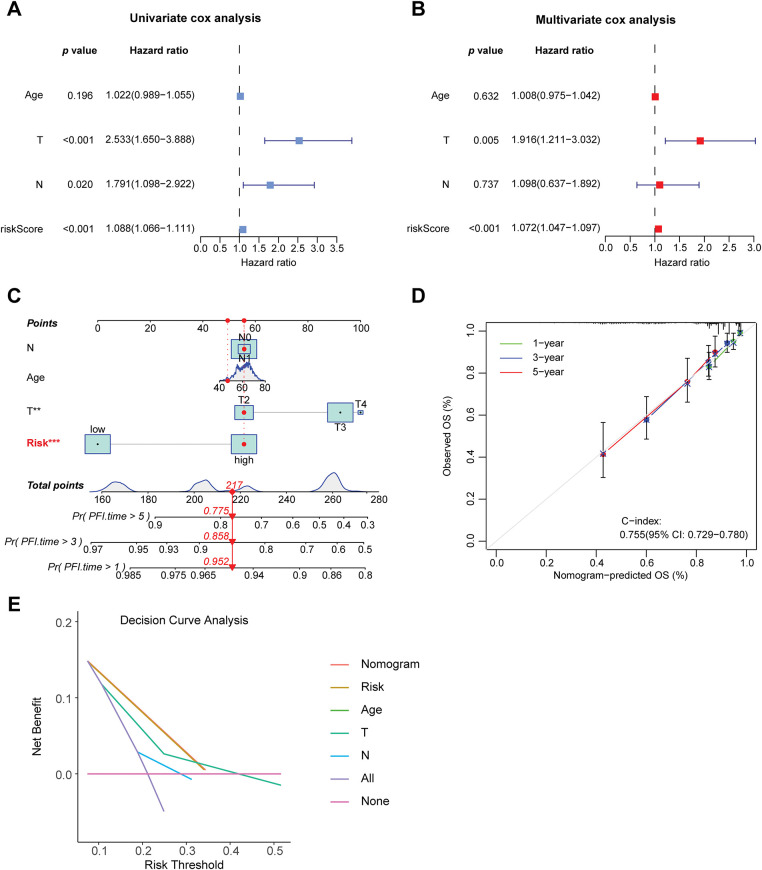
Construction of the nomogram and decision curve. (**A**) Forest plot of univariate COX regression analysis. (**B**) Forest plot of multivariate COX regression analysis. (**C**) Constructing a nomogram to predict patients’ progression-free survival at 1, 3, and 5 years in conjunction with clinical information. (**D**) Calibration curve of the nomogram. (**E**) Results of decision curve analysis. **p < 0.01, ***p < 0.001

To evaluate whether clinical characteristics such as age, T stage, N stage, and risk score can serve as independent prognostic factors, this study then conducted univariate and multivariate COX regression analyses. The univariate COX analysis results demonstrated that T stage, N stage, and risk score were associated with patient prognosis (*p* < 0.05) (Fig. 5A). The multivariate COX analysis results confirmed that the risk score serves as an independent predictor of patient prognosis, irrespective of other variables (*p* < 0.001) (Fig. 5B). To improve the predictive ability of the model by integrating clinical information, this study also integrated the risk score with multiple clinical variables. A nomogram was constructed using the Cox proportional-hazards model and was calibrated and validated (Fig. 5C,D). By summing up the scores of the corresponding factors in the nomogram, the probabilities of 1-year, 3-year, and 5-year PFS of patients could be obtained. The value of the concordance index (C-index) can quantify the discriminatory ability of the model. The C-index value of the nomogram constructed in this study was 0.755, showing that this nomogram has good predictive ability. Finally, we performed decision curve analysis (Fig. 5E), which illustrated that the predictive capability of the prognostic model and the nomogram is superior to that of the clinical features applied in isolation.

### 3.5 Analysis of the Biological Characteristics Associated with Risk Stratification

**Figure 6:**
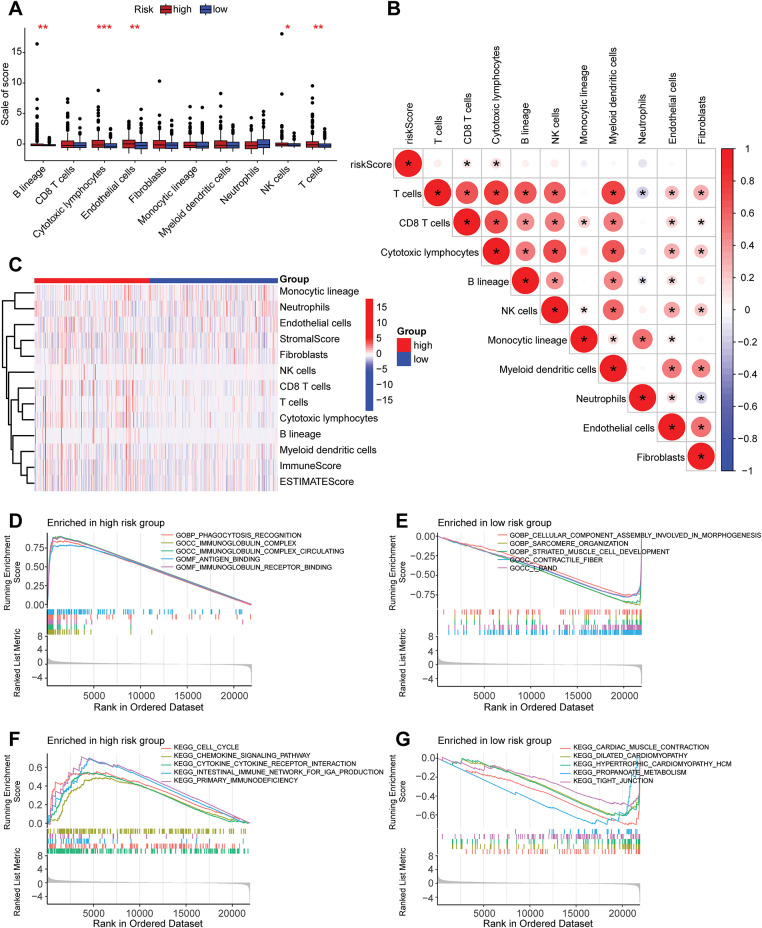
Analysis related to immune cells and GSEA analysis of risk stratification. (**A**) Differential analysis of ten distinct immune cell types between high-risk and low-risk groups, with the *x*-axis representing the names of immune cells; ***p < 0.001, **p < 0.01, *p < 0.05. (**B**) A correlation heatmap depicting the relationship between risk scores and ten immune cell types. *p < 0.05. (**C**) A heatmap illustrating the differences in cell types and scores between high-risk and low-risk groups. (**D**) GSEA analysis of GO gene sets in the high-risk group. (**E**) GSEA analysis of GO gene sets in the low-risk group. (**F**) GSEA analysis of KEGG gene sets in the high-risk group. (**G**) GSEA analysis of KEGG gene sets in the low-risk group. GSEA: Gene Set Enrichment Analysis. GO: Gene Ontology. KEGG: Kyoto Encyclopedia of Genes and Genomes

A correlation analysis was performed between the risk score and immune checkpoint genes to evaluate the potential utility of the developed prognostic model for immunotherapy (Supplementary Fig. S1). The risk score exhibited a positive correlation with POLE2, FEN1, MCM6, POLD3, MSH2, and MSH6, while showing a negative correlation with TAGLN. A differential analysis of the expression of 12 immune checkpoint genes in high- and low-risk group samples was conducted (Supplementary Fig. S2). It was found that the expression levels of genes such as CD274, FAP, LOXL2, and PDCD1 were higher in the high-risk group samples than in the low-risk group (*p* < 0.05), while the expression of TAGLN was higher in the low-risk group samples than in the high-risk group, indicating the research potential of these genes as targets for prostate cancer immunotherapy. Further differential analysis of immune cell infiltration in the high- and low-risk groups was carried out (Fig. 6A,C). The infiltration levels of NK cells, cytotoxic lymphocytes, and B cells in high-risk group samples were significantly elevated compared to those in the low-risk group. The correlation analysis results between the risk score and immune cells (Fig. 6B) indicated a positive correlation between these immune cells and the risk score.

GSEA is a statistical method used to identify significant changes in functionally related gene sets within gene expression data. In this study, GSEA was conducted based on GO and KEGG for high-risk and low-risk groups, aiming to uncover differences in gene functions and metabolic pathways between samples of varying risk levels. The GO-based GSEA analysis revealed that the gene functions significantly enriched in the high-risk group were primarily associated with immune response and antigen recognition, including processes such as phagocytosis recognition, antigen binding, and immunoglobulin receptor binding (Fig. 6D). Conversely, the low-risk group exhibited significant enrichment in gene functions related to cellular structure and muscle development (Fig. 6E). Genes in the high-risk group were notably involved in immune system activation and antibody-mediated immune responses, such as B cell function and pathogen recognition, suggesting that an active immune microenvironment may be linked to disease progression or poor prognosis. In contrast, genes in the low-risk group were concentrated on cellular structural stability, muscle tissue development, and contraction functionality, potentially related to tissue repair, differentiation, or metabolic homeostasis, reflecting favorable prognostic characteristics. The KEGG-based GSEA analysis indicated that the biological pathways mainly enriched in the high-risk group were predominantly associated with immune and inflammatory pathways, such as chemokine signaling pathways and cell proliferation-related pathways (Fig. 6F). The low-risk group, on the other hand, showed significant enrichment in biological pathways related to propanoate metabolism (Fig. 6G), a short-chain fatty acid metabolic pathway involved in energy supply and anti-inflammatory processes, suggesting that metabolic balance may inhibit malignant phenotypes.

**Figure 7:**
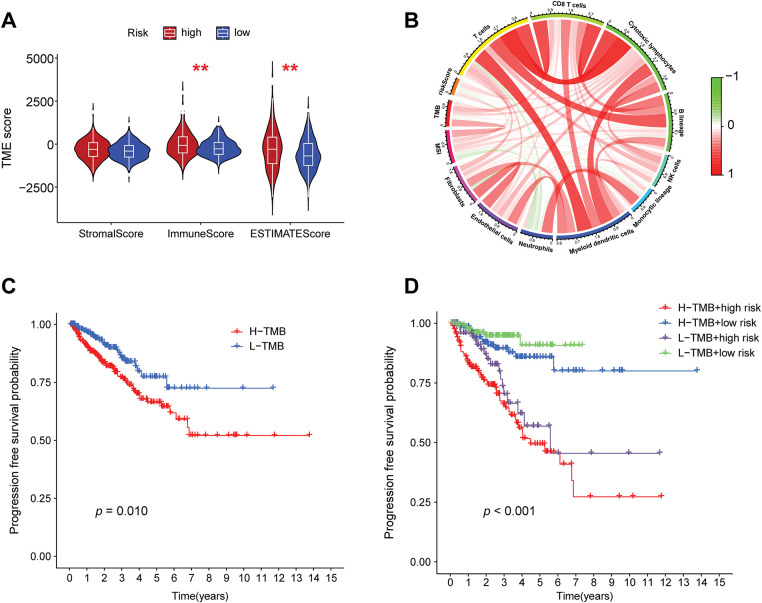
Correlation analysis of risk grouping with tumor microenvironment and tumor mutation burden. (**A**) Differences in the stromal score, immune score, and composite score of the tumor microenvironment between the high- and low-risk groups, **p < 0.01. (**B**) A chord diagram showing the correlations among the risk score, tumor mutation burden, microsatellite instability, and immune cell infiltration. (**C**) Progression-free survival (PFS) analysis of the high- and low-tumor mutation burden groups. (**D**) PFS analysis of the combined grouping of high/low tumor mutation burden and risk groups

To further explore the differences in tumor microenvironments between high-risk and low-risk groups, this study integrated grouping and TME scoring data, employing combined violin and box plots to visually illustrate the distribution differences in stroma, immune, and composite scores across different risk groups (Fig. 7A). It was observed that the high-risk group exhibited higher immune scores compared to the low-risk group, indicating that high-risk samples possess more active immune infiltrating components, potentially associated with a complex microenvironment or resistance mechanisms. Correlation analysis of the tumor microenvironment with tumor mutational burden (TMB) (Fig. 7B) revealed that various immune cell infiltrations positively regulate TMB. PFS analysis, as illustrated in Fig. 7C, indicated that patients with low TMB levels exhibited significantly improved PFS relative to those with high TMB levels. Subsequently, this study combined these findings with risk grouping to plot PFS curves for the four groups (Fig. 7D), which exhibited statistically significant differences, indicating that the integration of risk scores can enhance prognostic stratification capabilities.

### 3.6 The Function of Prognostic Gene AK5 in PCa by Bioinformatic Analysis

**Figure 8:**
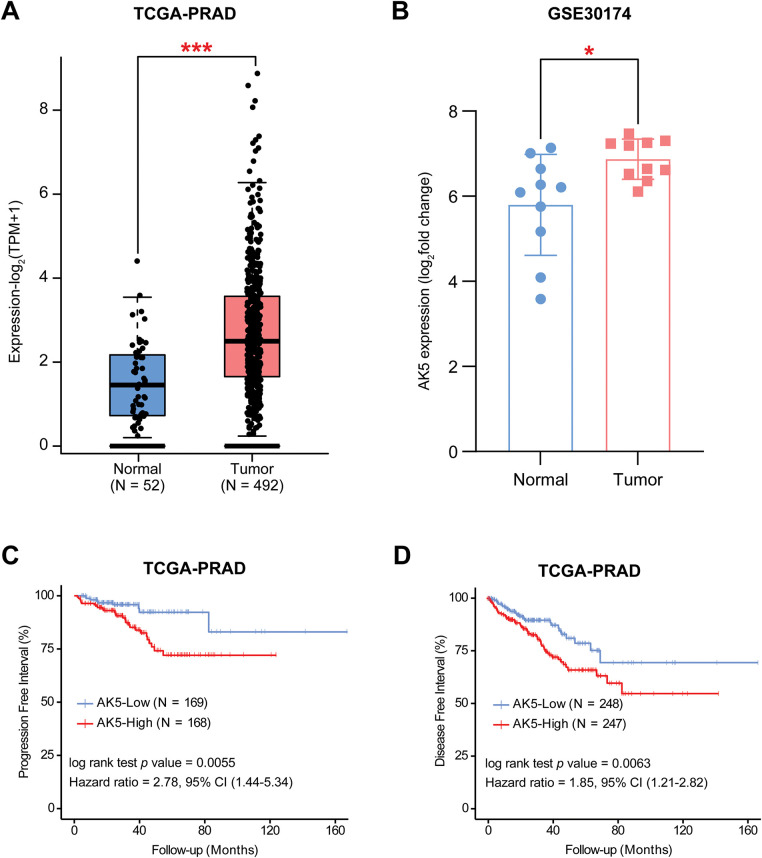
Differential expression analysis and survival analysis results of AK5 in public databases. (**A**) Box plot illustrating the expression differences of AK5 between tumor and normal samples in the TCGA-PRAD cohort. (**B**) Box plot depicting the expression differences of AK5 between tumor and normal samples in the GEO-GSE30174 dataset. (**C**) Kaplan-Meier curve for progression-free survival generated using prostate cancer samples from the TCGA-PRAD cohort. (**D**) Kaplan-Meier curve for disease-free survival generated using prostate cancer samples from the TCGA-PRAD cohort. All data are expressed as means ± standard deviation and *p* values were calculated using unpaired *t*-test (**A**, **B**) and Kaplan-Meier log-rank test (**C**, **D**). *p < 0.05, ***p < 0.001

The risk score model constructed in this study consists of 10 genes. After searching the published literature, it was found that there is limited study on the role of AK5 in PCa. The AK5 (Adenylate kinase 5) gene belongs to the adenylate kinase family. Its core function is to catalyze the reversible transformation of ATP and AMP and maintain the intracellular adenylate energy homeostasis [23,24]. In this study, the difference in AK5 expression levels was analyzed in tumors and normal samples of the TCGA-PRAD cohort (Fig. 8A), and found that AK5 expression in tumors was obviously higher than that of normal tissues. The same results were obtained for the analysis of the differences between AK5 in tumors and normal samples in GEO-GSE30174 dataset (Fig. 8B). At the same time, in the prostate cancer cohort of TCGA, progression-free survival was obviously better than that of patients with high expression (Fig. 8C), and the analysis results of disease-free survival also supported the same conclusion (Fig. 8D).

**Figure 9:**
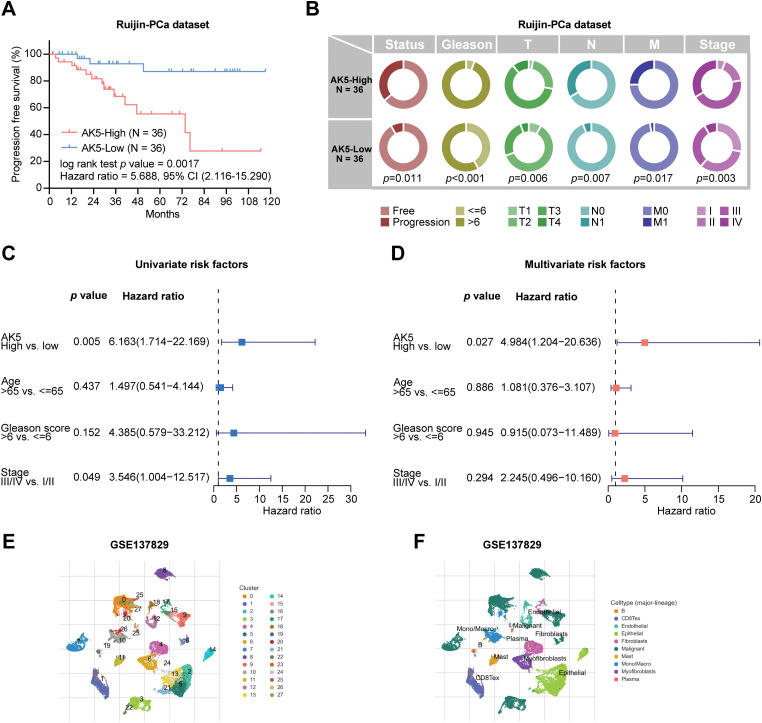

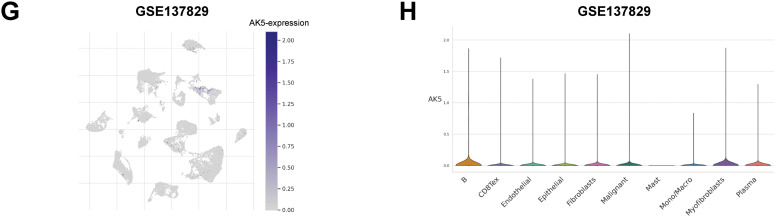
Analysis of AK5 in the prostate cancer cohort at Ruijin Hospital and single-cell transcriptome data. (**A**) Kaplan-Meier curve of progression-free survival based on the Ruijin prostate cancer cohort. (**B**) Pie chart illustrating the correlation between AK5 expression levels and various clinical variables. (**C**) Univariate COX analysis based on the Ruijin prostate cancer dataset. (**D**) Multivariate COX analysis based on the Ruijin prostate cancer dataset. (**E**) UMAP visualization of cell subpopulations clustered from the single-cell data of the GSE137829 dataset. (**F**) UMAP representation of the distribution of different cell types. (**G**) Expression profile of the AK5 gene within the cell subpopulations. (**H**) Expression differences of AK5 across various cell subpopulations

The relationship between AK5 expression levels and progression-free survival in patients was subsequently analyzed in the PCa cohort at Ruijin Hospital (Fig. 9A). Patients exhibiting elevated AK5 expression demonstrated a worse prognosis. The analysis, when integrated with the patients’ clinical information, revealed a correlation between the expression level of AK5 and various clinical features, including Stage and TNM staging (Fig. 9B). Univariate COX regression analysis utilizing the Ruijin prostate cancer dataset indicated a correlation between AK5 expression level and patient prognosis (Fig. 9C). Furthermore, multivariate COX regression analysis demonstrated that AK5 expression level function as an independent prognostic factor (Fig. 9D). To further analyze the expression of AK5 in various cells of tumor tissues, single-cell transcriptome data of prostate cancer were collected from the GEO-GSE137829 dataset. The expression profile of the AK5 gene in cell subsets was plotted (Fig. 9E–H). It was found that AK5 was expressed in multiple cell subsets, with relatively high expression in myofibroblasts.

### 3.7 The Experimental Study of AK5 in PCa

**Figure 10:**
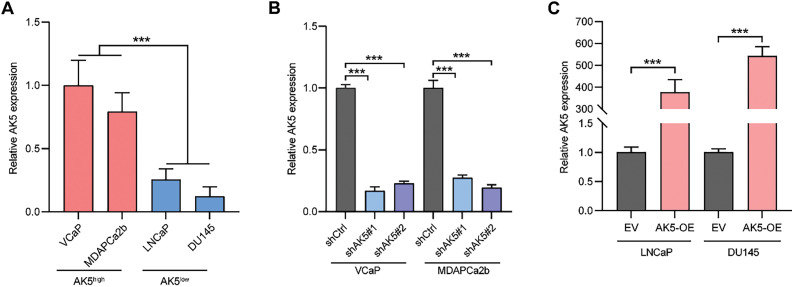

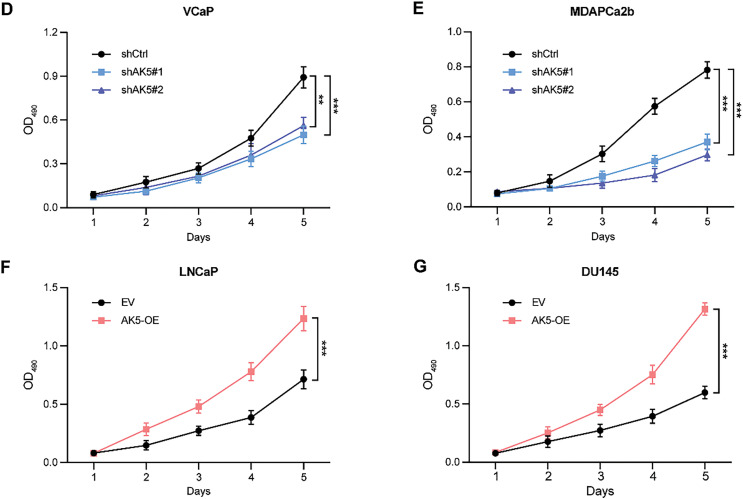
After conducting knockdown and overexpression of AK5, MTS experiment was used to detect the impact of AK5 on the proliferation of prostate cancer cells. (**A**) The relative expression levels of AK5 mRNA in four untreated prostate cancer cell lines (n = 5). (**B**) RT-qPCR validation of the knockdown levels of AK5 by lentivirus in the VCaP and MDAPCa2b cell lines, with GAPDH as the internal control gene. (**C**) RT-qPCR validation of the overexpression levels of AK5 by lentivirus in the LNCaP and DU145 cell lines, with GAPDH as the internal control gene. (**D**) The change in absorbance at 490 nm in the VCaP cell line after the knockdown of AK5 compared to the unmodified cell line (n = 5). (**E**) The change in absorbance at 490 nm in the MDAPCa2b cell line after the knockdown of AK5 compared to the unmodified cell line (n = 5). (**F**) The change in absorbance at 490 nm in the LNCaP cell line after the overexpression of AK5 compared to the non-overexpressing cell line (n = 5). (**G**) The change in absorbance at 490 nm in the DU145 cell line after the overexpression of AK5 compared to the non-overexpressing cell line (n = 5). All data are expressed as means ± standard deviation and *p* values were calculated through unpaired *t*-test (**A**, **C**), one-way analysis of variance (ANOVA) with Tukey’s multiple comparison test (**B**), and two-way repeated-measures ANOVA with Sidak’s posthoc test (**D**–**G**). **p < 0.01, ***p < 0.001. RT-qPCR: Reverse transcription quantitative polymerase chain reaction

The preceding analysis indicates that the AK5 gene may play an oncogenic role in PCa. This study selected four PCa cell lines to further explore the role of AK5 through both *in vitro* and *in vivo* experiments. After measuring the AK5 mRNA levels in the four cell lines, it was found that VCaP and MDAPCa2b cell lines exhibited higher expression, while LNCaP and DU145 cell lines showed lower expression levels (Fig. 10A). Consequently, the first two cell lines were chosen to establish the AK5 knockdown group, while the latter two were utilized for AK5 overexpression. The results of RT-qPCR demonstrated successful knockdown of AK5 expression in VCaP and MDAPCa2b cell lines, and successful overexpression of AK5 in LNCaP and DU145 cell lines (Fig. 10B,C).

In MTS cell proliferation assays, all four cell lines were utilized, and the changes in absorbance at 490 nm were monitored over a continuous period of five days. It was observed that the absorbance changes in the VCaP and MDAPCa2b cell lines, following AK5 knockdown, were significantly reduced compared to the non-knockdown counterparts (Fig. 10D,E), indicating a decline in the proliferative capacity of the tumor cells post-knockdown of AK5. Conversely, the LNCaP and DU145 cell lines exhibited a marked increase in absorbance changes following AK5 overexpression compared to their non-overexpressing controls (Fig. 10F,G), suggesting an enhancement in the proliferative capacity of the tumor cells after AK5 overexpression.

**Figure 11:**
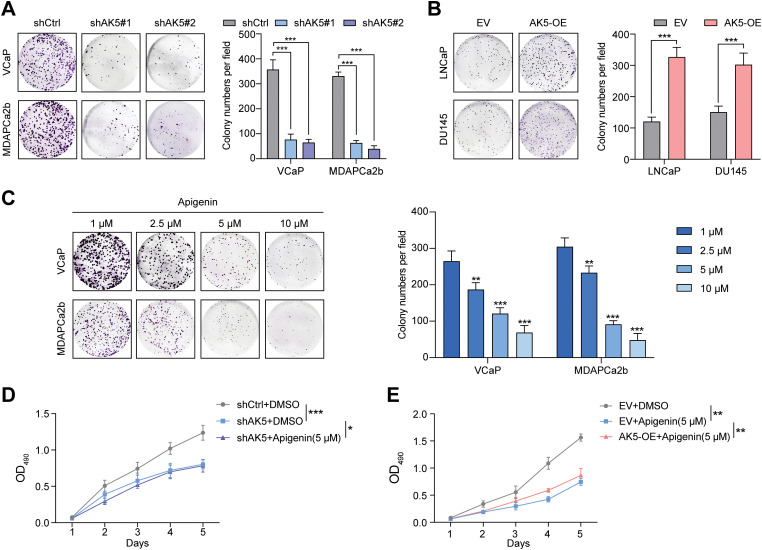

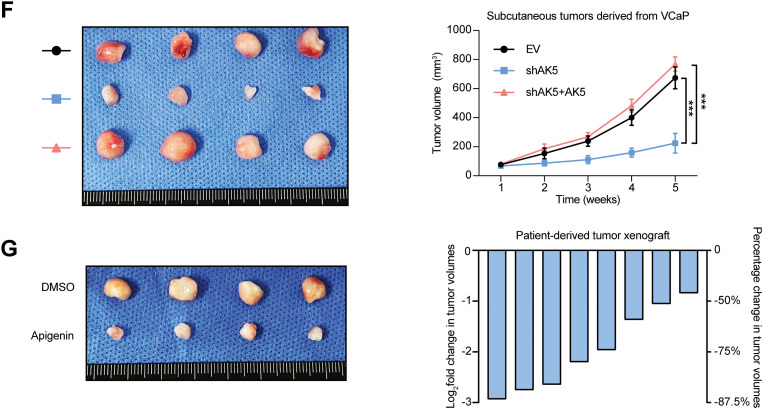
Identification of the role of AK5 in prostate cancer through *in vitro* and *in vivo* experiments. (**A**) Representative clonal formation assay results of the changes in the proliferative capacity of two prostate cancer (PCa) cell lines following the knockdown of AK5 and the quantitative results (n = 3). (**B**) Representative clonal formation assay results of the changes in the proliferative capacity of two prostate cancer cell lines after the overexpression of AK5 and the quantitative results (n = 3). **(C**) The representative results of colony formation assays in two prostate cancer cell lines treated with varying concentrations of Apigenin and the quantitative results (n = 3). (**D)** The impact of AK5 knockdown on the effects of Apigenin on cell proliferation in the VCaP cell line (n = 3). (**E)** The impact of AK5 overexpression on the effects of Apigenin on cell proliferation in the LNCaP cell line (n = 3). (**F)** The impact of AK5 knockdown on tumor growth (n = 5). (**G)** The effects of Apigenin on tumors derived from prostate cancer patient-derived xenograft (PCa-PDX) models. All data are expressed as means ± standard deviation and *p* values were calculated through unpaired *t*-test (**B**), one-way analysis of variance (ANOVA) with Tukey’s multiple comparison test (**A**,**C**), and two-way repeated-measures ANOVA with Sidak’s posthoc test (**D**–**F**). *p < 0.05, **p < 0.01, ***p < 0.001

To validate these conclusions, we conducted colony formation assays with the four cell lines. The analysis revealed a significant decrease in the number of colonies formed by the two cell lines with AK5 knockdown (Fig. 11A), whereas the colonies formed by the two cell lines with AK5 overexpression showed a significant increase compared to the control group (Fig. 11B). The findings collectively validate the oncogenic function of AK5.

Subsequently, subcutaneous xenograft tumor model in nude mice was established using the VCaP cell line. The experiment was divided into three groups. Starting from the 4th day after inoculation, the volume was measured every 7 days for a continuous period of 5 weeks. The volume was recorded, and finally, the tumors were excised. The analysis results (Fig. 11F) showed that knocking down AK5 significantly inhibited tumor growth, while rescuing AK5 expression after knockdown could eliminate this inhibitory effect.

Finally, this study employed Apigenin, which was reported to have anti-tumor effects [25]. Colony formation assays indicated that inhibiting AK5 could reduce the proliferative ability of tumor cells (Fig. 11C). The patient-derived xenograft (PDX) model also demonstrated that the tumor volume was obviously reduced after the application of the Apigenin (Fig. 11G). We also utilized cell lines with knockdown and overexpression of AK5 and re-administered apigenin to evaluate its impact on the proliferative capacity of tumor cells. The findings from the MTS cell proliferation assay indicated that following the knockdown of AK5, the application of Apigenin only slightly inhibited the proliferative capacity of the cells (Fig. 11D). And overexpression of AK5 can counteract some of the inhibitory effects of Apigenin (Fig. 11E). This suggests that the inhibitory effect of Apigenin is at least partially mediated through the inhibition of AK5. Based on the above experimental results, it is clear that AK5 plays a pro-oncogenic role in prostate cancer.

## 4 Discussion

Nowadays, the role of immune and metabolic reprogramming in tumorigenesis has garnered significant attention [26–29]; nonetheless, the precise mechanisms and clinical applications of these processes in prostate cancer require further investigation [30–32]. This study integrates various transcriptomic datasets and functional experiments to construct subtype and prognostic models of PCa based on immune and metabolic genes. Furthermore, experimental evidence has preliminarily confirmed the oncogenic role of AK5 in prostate cancer.

The occurrence and progression of PCa are closely associated with immunological factors. Studies indicate that the infiltration of CD8^+^ T cells in certain prostate cancer tissues is significantly associated with adverse prognostic outcomes in patients [33–35]. Compared with normal prostate tissues, there is also more infiltration of B cells in cancer tissues, and it is related to the degree of aggressiveness [36,37]. There are also macrophage populations with M1 and M2 phenotypes in the TME of PCa [38]. Previous studies have indicated that an abnormally increased proportion of M2 macrophages in tumor tissues is related to early biochemical recurrence of prostate cancer [39]. M1 macrophages also play an indirect role by influencing the polarization of M2 macrophages through various cytokines [40]. Myeloid-derived suppressor cells (MDSC) in the TME also participate in the regulation of tumor cell drug resistance. Through costimulatory interactions with mast cells, they mediate tumor cell immune escape [41]. MDSC exhibits a negative correlation with the extent of T cell infiltration in prostate tumor tissues. Eliminating MDSC can promote anti-tumor responses [42]. The mechanism of action of some drugs also includes regulating the expression of immune-suppressive genes in MDSC [43]. The genomic features of tumors significantly influence the tumour microenvironment (TME). In different regions and among different patients, different immunogenomic subtypes of prostate cancer can often be detected. Based on the differences in immunogenomics, different immunotherapies can be developed to exert targeted effects respectively, providing benefits for more precise treatment. Research focused on immune-related genomes and transcriptomes holds significant potential for elucidating the mechanisms underlying the onset and progression of PCa, identifying novel biomarkers, and establishing new therapeutic targets.

PCa is closely associated with metabolic factors, and the metabolic characteristics of tumor cells along with specific metabolic pathways have become a focal point of research in recent years [44]. The exploration of metabolic reprogramming provides theoretical support for various novel anti-cancer therapies. Studies have indicated that metabolic pathways such as the tricarboxylic acid (TCA) cycle are excessively activated in prostate tumor cells [45], with enhanced glycolysis contributing to energy production for tumor cell proliferation [46]. As the disease progresses and castration resistance develops, tumor cells actively utilize lipids and glutamine as energy substrates that confer adaptive advantages. In normal prostate epithelial cells, elevated zinc levels inhibit aconitase, thereby suppressing the TCA cycle, which is marked by the active production and secretion of citrate. Citrate serves as a significant element of prostatic fluid and is essential for the preservation of sperm viability. In contrast, prostate tumor cells exhibit low levels of zinc-inhibited aconitase, leading to reduced citrate secretion and increased expression of glycolysis-related transport proteins and enzymes, significantly altering the metabolic landscape of the prostate [47], a feature that is even more pronounced in advanced tumors. Additionally, glutamine is vital for the proliferation and growth of prostate epithelial cells, with increased glutamine uptake and upregulation of glutaminase observed in prostate tumor cells [48], enhancing glutamine metabolism. Moreover, lipid substances are also involved in the aberrant metabolic processes of PCa [49–51]. Research indicates that CD36-mediated fatty acid uptake is essential for generating lipid oncogenic signals in prostate tumor tissues. High CD36 expression correlates positively with reduced survival rates and the progression of metastasis [52]. The abnormal accumulation of lipid droplets in prostate tumor cells is commonly observed and contributes to tumor cell survival and growth. This phenomenon is also linked to drug resistance, likely due to the retention of hydrophobic therapeutic agents, which reduces their efficacy [53].

The malignant transformation of normal prostate cells occurs through a series of biological changes. Mutations in metabolism-related oncogenes or tumor suppressor genes play a significant role [54,55]. For instance, fatty acid synthase (FASN) is a crucial enzyme involved in the synthesis of long-chain fatty acids and serves as a critical target for *de novo* lipogenesis, being overexpressed in malignant prostate tumors compared to normal prostate tissues, and is highly correlated with increased invasiveness, the evolution of castration resistance, and bone metastasis [56]. Studies have also described the significant role of androgens and androgen receptors in the metabolic regulation of prostate cancer, involving multiple processes including glycolysis, glutamine metabolism, and lipid and cholesterol metabolism [57].

Furthermore, some studies have demonstrated a high correlation between immune cells and the metabolic processes in prostate cancer [58]. However, the metabolism of circulating immune cells involves a series of complex regulatory processes [59,60], and there are few studies that combine immune and metabolic genes to investigate prostate cancer. Recent studies have explored the correlation between specific immune or metabolic process-related genes and the prognosis of PCa [61]. Within the immune prognostic models, there are those based on macrophage-related genes [20,62], NK/T cell communication [63], regulatory T cells specific genes [64], seven immune-inflammatory indices [65], and 14 crucial monocyte-related genes [66]. The metabolic gene prognostic model was on the basis of fatty acid metabolism-related genes [67,68].

On the basis of the differential analysis of immune metabolic genes from TCGA, this study successfully classified prostate cancer into three molecular subtypes (Cluster 1, 2, and 3), with patients in the Cluster 1 subtype exhibiting the most favorable prognosis and significant immune cell infiltration (such as monocytes and cytotoxic lymphocytes). This finding is consistent with existing research indicating a close correlation between the degree of immune infiltration and improved survival in PCa patients [69]. However, in the subsequent construction of prognostic models, the high-risk group demonstrated higher immune score yet exhibited a poorer prognosis. This paradox may be attributed to the complicacy of the immune microenvironment. This study identified a notable increase in the expression of CD274 and PDCD1 within the high-risk group, indicating that the activation of immune checkpoint pathways may contribute to immune evasion and promote disease progression [70]. Furthermore, the significant enrichment of the propionate metabolic pathway in the low-risk group further supports the potential role of metabolic homeostasis in tumor suppression. Short-chain fatty acids demonstrate anti-inflammatory and anti-tumor properties by regulating histone deacetylase activity or modulating gut microbiota composition [71]; however, their specific mechanisms in PCa require further validation.

Our study identifies ten prognostic genes through LASSO regression and multifactorial COX analysis. The constructed model demonstrates robust predictive efficacy in both TCGA and GEO datasets. The joint analysis of the risk score and TMB further enhances prognostic stratification capabilities. Although high TMB is generally considered to correlate positively with responses to immunotherapy, this research suggests that patients with high TMB in PCa exhibit poorer prognoses. This finding suggests that genomic instability may be accompanied by the accumulation of driver mutations, such as deletions in TP53 and PTEN, which could lead to therapeutic resistance [72]. Additionally, single-cell transcriptomic analysis reveals that AK5 is significantly expressed in myofibroblasts, suggesting its potential role in modulating tumor-stroma interactions that influence the metabolic state of the microenvironment. For instance, extracellular matrix components secreted by myofibroblasts, such as collagen, may promote tumor invasion through integrin signaling pathways [73], while AK5-mediated energy metabolism reprogramming may further exacerbate this process.

Previous studies have indicated that AK5 is downregulated in neurodegenerative diseases and is associated with disturbances in brain energy metabolism. Conversely, it is overexpressed in tumors such as glioblastoma, potentially driving proliferation by enhancing the energy supply to cancer cells. The protein structure of AK5 contains a typical adenylate kinase functional domain and is regulated by the AMPK pathway in response to energy stress [74]. Aberrant expression of AK5 is closely linked to metabolic imbalance, neuroprotection, and tumor progression [75], suggesting its potential as a biomarker or therapeutic target for energy metabolism-related diseases [23]. Our study reveals the oncogenic role of AK5 in prostate cancer. Functional experiments confirm that the knockdown of AK5 obviously inhibits the prostate cancer cells’ proliferation and clonogenic ability, while its overexpression promotes malignant phenotypes. In animal experiments, the knockdown of AK5 markedly affected the development of xenograft tumors in nude mice and this inhibitory effect could be reversed by restoring expression, further validating the oncogenic dependency on AK5. Additionally, the Apigenin demonstrated antitumor effects *in vitro* and in PDX models, providing experimental evidence for its clinical translation.

Despite the achievements of this section of the study, several limitations remain. The construction of the prognostic model relies on retrospective data from public databases, necessitating validation of its clinical applicability in prospective cohorts. The metabolic interactions among different cellular subpopulations within the immune microenvironment may influence the interpretative power of the prognostic model, warranting further investigation through spatial transcriptomics or metabolic imaging techniques. Furthermore, the specific pathways through which AK5 exerts its effects have not been fully elucidated, requiring a combined approach with metabolomics to further explore how it influence metabolic network remodeling and signal transduction. Further research can concentrate on assessing tumor cell metabolism following knockdown or overexpression of the gene AK5. Apigenin is a known multi-target flavonoid with pleiotropic biological effects and may act partially via modulation of AK5. There is still a limitation on the specificity of Apigenin’s targeting of AK5 and it is necessary for the development of inhibitors specifically targeting AK5.

## 5 Conclusion

Our research constructs a relatively robust prognostic model on the basis of immune and metabolic genes, which combines multiple transcriptome data and functional experiments. We preliminarily validate the pro-oncogenic mechanism of AK5. The research results provide insights for the exploration of new therapeutic targets for PCa. Future research needs to further analyze the spatiotemporal dynamics of metabolic reprogramming and its interaction network with the immune microenvironment to promote its clinical translation in PCa.

## Supplementary Materials



## Data Availability

The datasets presented in our study can be found on public websites mentioned in the methods. The software code involved in data processing can be obtained by contacting the corresponding author.

## Supplementary Materials

The supplementary materialis available online at https://www.techscience.com/doi/10.32604/or.2025.066783/s1.

## List of abbreviations

ADT: Androgen deprivation therapy
AUC: Area under the curve
CRPC: Castration-resistant prostate cancer
C-index: Concordance index
EV: Empty vector
FDR: False Discovery Rate
GEO: Gene Expression Omnibus
GO: Gene Ontology
GSEA: Gene Set Enrichment Analysis
HR: Hazard ratio
KEGG: Kyoto Encyclopedia of Genes and Genomes
LASSO: Least Absolute Shrinkage and Selection Operator
MDSC: Myeloid-derived suppressor cells
MTS: 3-(4,5-dimethylthiazol-2-yl)-5-(3-carboxymethoxyphenyl)-2-(4-sulfophenyl)-2H-tetrazolium
NMF: Non-negative Matrix Factorization
OS: Overall Survival
PCa: Prostate cancer
PDX: Patient-derived xenograft
PFI: Progression-free interval
PFS: Progression-free survival
ROC: Receiver operating characteristic
RT-qPCR: Reverse transcription quantitative polymerase chain reaction
TCGA: The Cancer Genome Atlas
TMB: Tumor mutational burden
TME: Tumor microenvironment
