# Supplementary material for: Construction of a Prognostic Model of Prostate Cancer Based on Immune and Metabolic Genes and Experimental Validation of the Gene AK5
Source: Oncol Res. 2025 Oct 22;33(11):3493–522. doi: 10.32604/or.2025.066783 (PMC12573196; doi:10.32604/or.2025.066783)
Supplement: Supplementary file 1 [file OncolRes-33-66783-s001.docx]

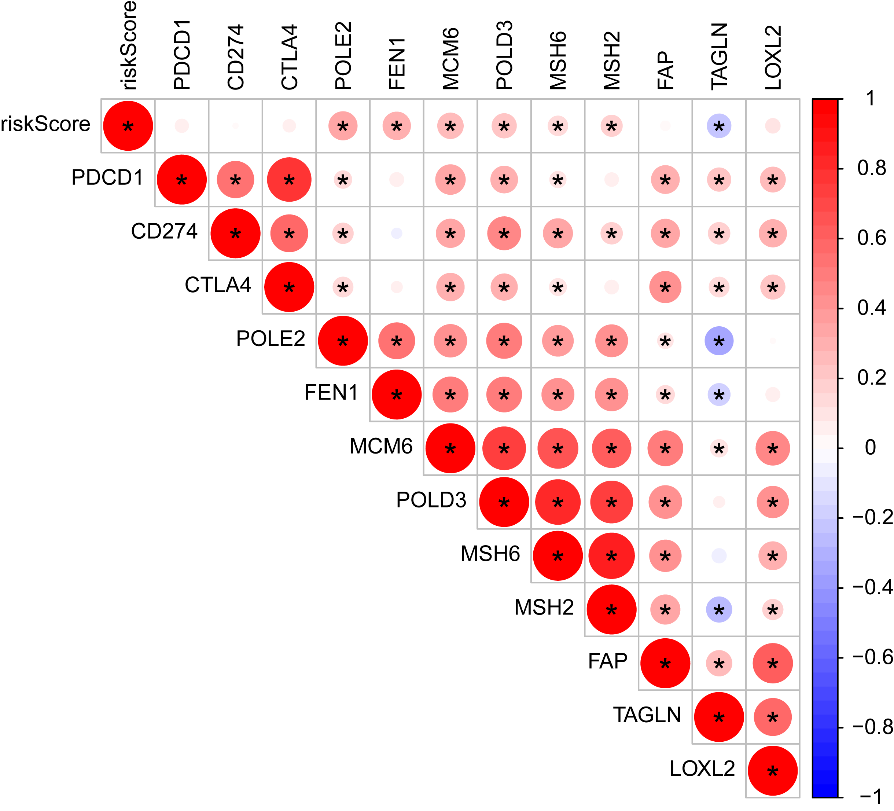


**Supplementary Figure S1. Correlation heatmap between Risk Score and immune checkpoint genes.** Red indicates a positive correlation, whereas blue signifies a negative correlation. The size of the circles corresponds to the strength of the correlation. **p* <0.05


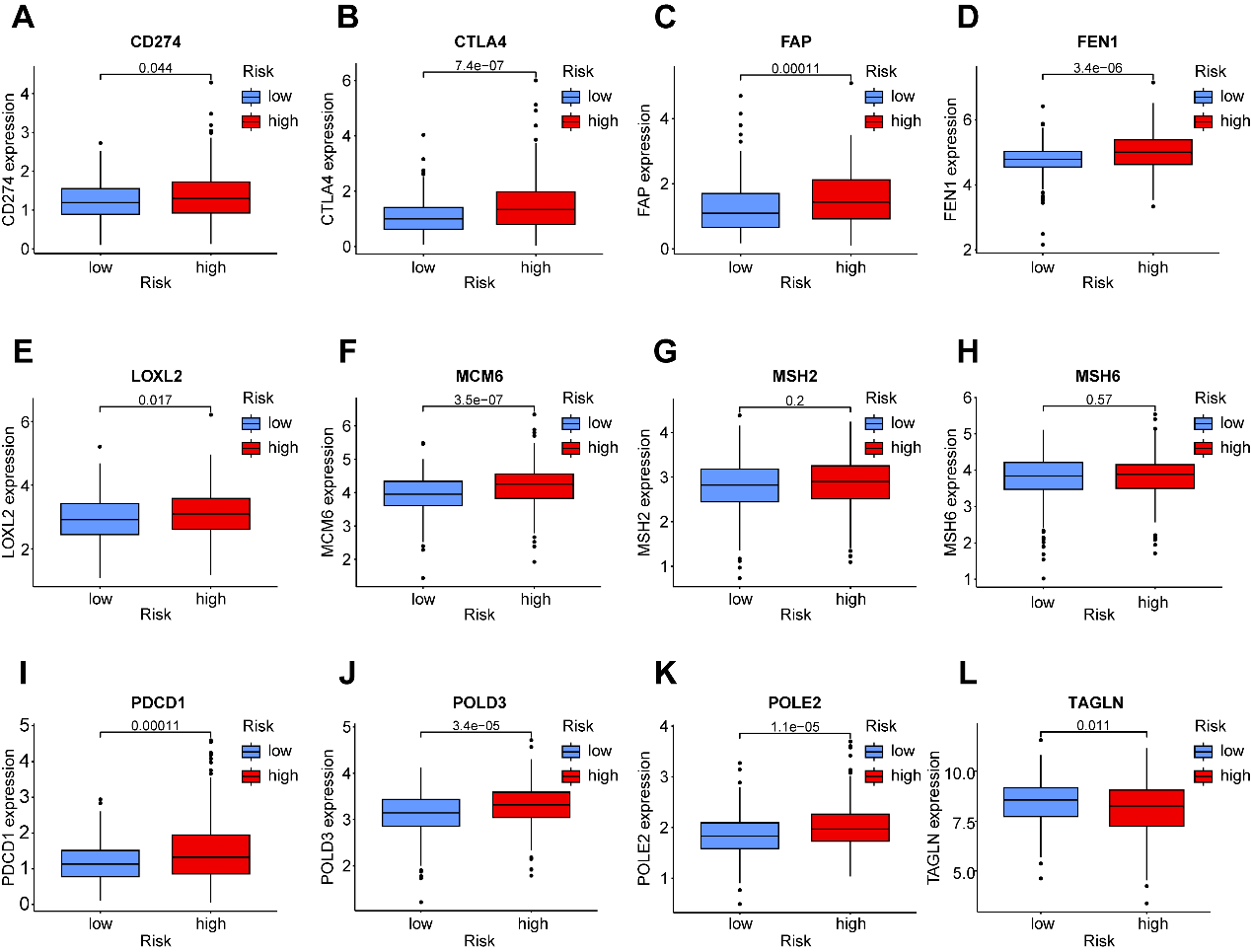


**Supplementary Figure S2. Differential expression of 12 immune checkpoint genes between high-risk and low-risk groups.** The vertical axis represents the names of immune checkpoint genes, while the horizontal axis denotes high-risk and low-risk groups.

**Supplementary Table S1.** Results of the Chi-square test for the distribution of clinical features in the training and testing datasets.

| **Covariates** | **Type** | **Total** | **Test** | **Train** | ***p*-value** |
| --- | --- | --- | --- | --- | --- |
| Age | <=65 | 354(71.23%) | 99(66.44%) | 255(73.28%) |  |
|  | >65 | 143(28.77%) | 50(33.56%) | 93(26.72%) | 0.15 |
| T | T2 | 187(37.63%) | 55(36.91%) | 132(37.93%) |  |
|  | T3 | 293(58.95%) | 91(61.07%) | 202(58.05%) |  |
|  | T4 | 10(2.01%) | 3(2.01%) | 7(2.01%) | 0.93 |
|  | unknow | 7(1.41%) | 0(0.00%) | 7(2.01%) |  |
| N | N0 | 345(69.42%) | 112(75.17%) | 233(66.95%) | 0.27 |
|  | N1 | 79(15.90%) | 20(13.42%) | 59(16.95%) |  |
|  | unknow | 73(14.69%) | 17(11.41%) | 56(16.09%) |  |
